# Ultrasound-Guided Transforaminal Injections of Platelet-Rich Plasma Compared with Steroid in Lumbar Disc Herniation: A Prospective, Randomized, Controlled Study

**DOI:** 10.1155/2021/5558138

**Published:** 2021-05-27

**Authors:** Zhen Xu, Shaoling Wu, Xiao Li, Cuicui Liu, Shengnuo Fan, Chao Ma

**Affiliations:** Department of Rehabilitation Medicine, Sun Yat-sen Memorial Hospital, Sun Yat-sen University, Guangzhou, Guangdong 510120, China

## Abstract

Transforaminal steroid injection is extensively used as a treatment in cases of herniated disc, but it is associated with complications. In comparison, platelet-rich plasma (PRP) injection has been used in musculoskeletal disorders and could be another option. This study is aimed at comparing the efficacy and safety aspects between ultrasound-guided transforaminal injections of PRP and steroid in patients who suffer from radicular pain due to lumbar disc herniation. In a randomized controlled trial, ultrasound-guided transforaminal injections of either PRP (*n* = 61) or steroid (*n* = 63) were administered to a total of 124 patients who suffer from radicular pain due to lumbar disc herniation. Patients were assessed by the visual analogue scale (VAS), pressure pain thresholds (PPTs), Oswestry disability index (ODI), and the physical function (PF) and bodily pain (BP) domains of the 36-item short form health survey (SF-36) before operation and 1 week, 1 month, 3 months, 6 months, and 12 months after operation. The rate and latency of F-wave were obtained before operation and 12 months postoperation. There was no statistical difference in terms of age and sex between both groups. Statistically significant improvements from the patients' data before operation to data obtained 1-month postoperation were observed in VAS, PPTs, ODI, and PF and BP of SF-36 in both groups and kept for 1 year. F-wave rate and latency were improved significantly at 1-year postoperation in both groups. Intergroup differences during follow-ups over a period of 1 year were not found to be significant in all the above assessment between the PRP and steroid groups. No complications were reported. The results showed similar outcome for both transforaminal injections using PRP and steroid in the treatment of lumbar disc herniation, suggesting the possible application of PRP injection as a safer alternative. The trial was registered in the Chinese Clinical Trial Registry (ChiCTR-INR-17011825).

## 1. Introduction

Low back pain is one of the most difficult conditions to manage for doctors, patients, and policymakers. Not only does it limit physical activity, life quality is also greatly reduced alongside additional social and economic burden. The point prevalence of low back pain is 12%, with its one-year prevalence being 38% and the lifetime prevalence being approximately 40% [1]. Aging population leads to the rising number of individuals affected by low back pain. Lumbar disc herniation has been identified as the common etiology of low back pain [2]. The treatments for lumbar disc herniation vary from conservative to surgical management, which include analgesics, traction, physical therapy, manipulation, and psychotherapy. However, not all patients are able to be relieved from pain through these treatments.

For over 30 years, epidural steroid injection has been widely used as a treatment for lumbar disc herniation [3, 4], with its effectiveness proven by multiple research [5–7]. It works in anti-inflammation, pain relief, and functional improvement. There are three routes for steroid injection: interlaminar, transforaminal, and caudal routes. The transforaminal route fared better than the other two because it could reach the targeted sites, namely, spinal nerve, anterior epidural space, and the dorsal root ganglion, to counteract the inflammation secondary to compression [8]. However, there are still concerns about the safety of epidural steroid injection. Based on literature, several complications related to epidural steroid injection have been pointed out, including neurotoxicity, pharmacologic effect of steroid (hypercorticism, adrenal suppression, and hyperglycemia), and neurologic injury [8, 9]. Besides, the contraindications of steroid use (allergy, diabetes, severe osteoporosis, pregnancy, severe hypertension, infection, etc.) limit the usage of epidural steroid injection.

Platelet-rich plasma (PRP), a biological product from the centrifugation of autologous blood with a high number of platelets in a small volume of plasma, has a positive effect on pain relief in some musculoskeletal diseases, especially osteoarthritis, tendinosis, and ligament tears [10]. PRP contains high concentration of growth factors (GFs) and cytokines that play important roles in anti-inflammatory, antiapoptotic, and proliferative effects on the neurons and fibroblasts [11]. Although the role of PRP in pain relief looks promising, the effect of transforaminal PRP injection in lumbar disc herniation with radicular pain remains unclear.

Henceforth, this study is aimed at investigating the efficacy and safety of ultrasound-guided platelet-rich plasma injections compared with steroid injections in treating lumbar disc herniation with radicular pain.

## 2. Materials and Methods

### 2.1. Study Design and Participant Recruitment

A prospective, randomized, and controlled study was carried out to compare the treatment of lumbar disc herniation with radicular pain with ultrasound-guided transforaminal steroid or PRP injections. This study was approved by the Ethics Committee of Sun Yat-sen Memorial Hospital, and the trial was registered with the Chinese Clinical Trial Registry (registration number: ChiCTR-INR-17011825). This investigation was conducted in accordance with the Declaration of Helsinki. 184 patients were assessed for eligibility based on the inclusion and exclusion criteria listed as follows.

Inclusion criteria are as follows: (1) aged 20-60 years; (2) low back pain with unilateral lower limb radicular pain, duration of more than 3 months; (3) posterolateral lumbar disc herniation of L4/L5 or L5/S1segment diagnosed by MRI or CT and consistent with the clinical symptoms and signs; (4) degree of pain (VAS) more than 5 and obvious symptoms and clinical signs of nerve root irritation; (5) had received conservative treatment, including physical therapy, manipulation, and nonmorphine treatment; (6) no symptoms of severe nerve damage including motor paralysis, muscle atrophy, and cauda equina syndrome; (7) had no history of spinal surgery.

Exclusion criteria are as follows: (1) infection; (2) had received prior injection treatment in the past 3 months, such as nerve root injection and caudal injection; (3) spinal tumors or tuberculosis; (4) multisegmental lumbar disc herniation, spinal deformity, or spinal stenosis; (5) not suitable for local injection; (6) allergic to the drug used in this study; (7) a history of drug abuse or oral anticoagulation; (8) pregnancy; (9) severe diabetes; (10) clinical diagnosis of heart disease, liver and kidney dysfunction, and hematological diseases; (11) abnormal psychological and cognitive disorders.

**Figure 1:**
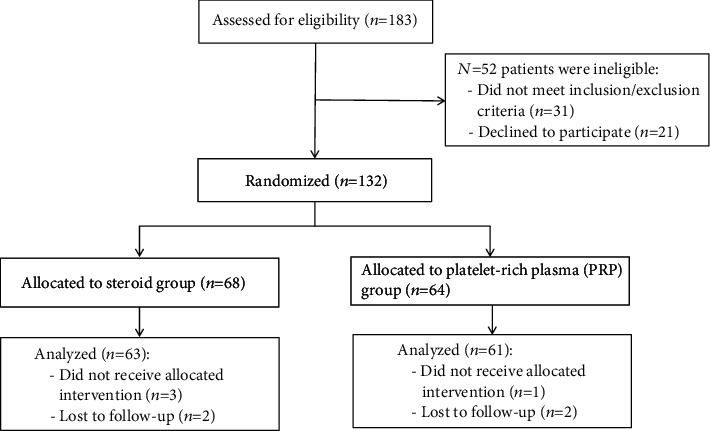
Flow diagram of enrolment, randomization, and analysis.

52 patients were not enrolled in this study (31 patients either did not meet the inclusion criteria or met the exclusion criteria, while the other 21 patients declined to participate). The randomization sequence was produced by a statistician, who did not contact with patients, using a random number generator. The other 132 patients who were enrolled were simply randomized at a ratio of 1 : 1 to 2 groups: the steroid group (control group, *n* = 68) and the PRP group (experimental group, *n* = 64). The randomization was performed by the nurse, who did not participate in the process of patient assessment, by opening the numbered sealed opaque envelop. A written informed consent was obtained from each patient before enrollment. During follow-ups over 1 year, 4 patients did not receive allocated intervention and 4 others did not follow through. Thus, only 124 patients were included for data analysis (steroid group *n* = 63, PRP group *n* = 61) (Figure 1).

### 2.2. Study Procedure

The patients who were enrolled to this study underwent physical examination, neurological examination, and laboratory tests. The operation was performed at the operating room in the rehabilitation medicine department of Sun Yat-sen Memorial Hospital.

The procedure of PRP preparation was as follows: 18 ml blood sample was drawn from the anterior elbow vein and mixed with 2 ml of 3.8% (*w*/*v*) sodium citrate. The blood sample was then centrifuged at 1600 rpm for 10 minutes at room temperature (RT, 23°C) under aseptic condition to divide the sample into 3 layers. The lower layers composed of red blood cells were subsequently removed. The remaining sample was transferred into a new centrifuge tube and was centrifuged again at 3200 rpm for 10 minutes at RT. 4 ml was collected from the lower part which contains PRP. 1 ml of this PRP was then sent for quantitative analysis of platelet count.

**Figure 2:**
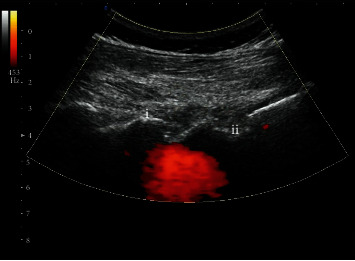
Ultrasound-guided transforaminal injection in out-of-plane approach. Ultrasound-guided transforaminal injection was performed, and the needle tip was positioned in the middle of (i) L5/S1 facet joint and (ii) sacral foramina. The red signal represented the spreading of drug in targeted epidural space.

The procedure of operation was as follows. An experienced physician performed the ultrasound-guided transforaminal injection using an ultrasonic device (Konica Minolta Medical & Graphic (Shanghai) Company limited, SONIMAGE HS1 PLUS, Tokyo, Japan) in out-of-plane approach. The patients were prepared in prone position with a pillow under their abdomen. The area of injection was disinfected. The sacral spinous process and the fifth lumbar spinous process transducer were identified when transducer placed longitudinally. The transducer in midline was moved laterally to recognize the lamina, transverse process, and facet joint. Then, the edge of the zygapophyseal joints was obtained when the transducer was moved back. The needle (22 G) was subsequently inserted into the skin using the out-of-plane approach upon determination of injection level to ensure that the needle tip was positioned in the middle of adjacent facet joints. After an inhalation test yielding negative results for cerebrospinal fluid and blood aspiration, the injection was administered (steroid group: 2 ml betamethasone+0.5 ml 0.9% sterile saline+0.5 ml 2% lidocaine; PRP group: 3 ml autologous PRP) (Figure 2). The detail of the ultrasound-guided out-of-plane injection can be found in this reference [12].

In the follow-up, some short-acting analgesics were given to patients when they felt obvious pain. The patients would be excluded from this study in follow-up when patients had any one of the following situations: (1) had overloaded pain which affects their life quality; (2) had symptom of motor paralysis, muscle atrophy, or cauda equina syndrome in the follow-up period; (3) transforaminal epidural injection failed to improve pain or function of patients during 3 months postoperation. Treatments including surgery or conservative treatments were given to those excluded patients.

### 2.3. Assessment and Outcome

The statistics of demographic characteristics, including gender and age, as well as baseline information of patients were collected upon admission of the patients. Baseline information was obtained before operation, which includes visual analogue scale (VAS), pressure pain thresholds (PPTs), the rate and latency of F-wave, Oswestry disability index (ODI), and physical function (PF) and bodily pain (BP) from the 36-item short form health survey (SF-36). Each patient was required to complete the same assessments as the baseline information (except F-wave rate and latency) at 1 week, 1 month, 3 months, 6 months, and 1 year postoperation. F-wave rate and latency were obtained only 1 year postoperation. All assessments were independently performed by two experienced blinded doctors.

The visual analogue scale (VAS) was a method to evaluate the degree of pain. A 10 cm line was used as an indicator whereby one end represents no pain, while the other end represents the most severe pain imagined. The patient was asked to indicate the point on the line which could represent the patient's pain level [13].

The pressure pain thresholds (PPTs) were measured by an algometry device (Pain Diagnostics and Thermography Corporation, Model PTH AF2, Great Neck, NY 11023) according to the procedure recommended by Fischer [14]. A plastic tip was placed at the paraspinal tenderness point in the segment with lumbar disc herniation. Detailed description can be found in our previous article [15].

The rate and latency of F-wave in the affected side were measured by electromyogram. The recording electrode was placed at the muscle belly of abductor halluces of the affected side; the reference electrode was placed at the tendon of abductor halluces; the stimulation electrode was placed at the tibial nerve behind the medial malleolus; the ground wire was placed at the ankle joint between the stimulation electrode and the recording electrode. At stimulation points of the affected side, 20 consecutive stimuli at the frequency of 1 Hz and the width of 0.2 ms were induced, obtaining records of the rate and latency of F-waves at the affected sides.

There were 10 items in the Oswestry disability index (ODI), including pain, individual function, and personal comprehensive function. The minimum score for each item is 0 (good state), whereas the highest score is 5 (poor state). The Oswestry disability index referred to the percentage of the sum of score from all 10 items out of 50.

The 36-item short form health survey (SF-36) consisted of 36 items, which includes one scale on health transition and 8 domains. The score for each domain ranges from 0 (poor health) to 100 (good health). The reliability, validity, and application of the Chinese version SF-36 have been proven [16]. In this study, two domains were recorded, namely, the physical function (PF) and bodily pain (BP) domains.

### 2.4. Statistical Analysis

With a sample size of 60 patients each group, we calculated that the study would have the power of 80% to detect a 0.9 difference at the significant improvement in VAS between groups from baseline to 6 months with the 2 standard deviation of the change in VAS. The mean and standard deviation were based on data from previous literature and our previous study [17, 18]. Besides, there was an increase of 15% in sample size in each group in case of the loss to follow-up. Two-sided *α* level was 0.05.

Data were analyzed using the SPSS 23.0 software. The continuous variables were expressed by mean ± standard deviation or medians (1st-3rd quartiles) depending on data distribution, while the discrete variable (such as sex) was described as *n* (%). The Shapiro-Wilk test was used to test the data distribution of the continuous variables. Continuous data before operation with nonnormal distribution was analyzed using the Mann–Whitney *U* test, whereas the discrete variable was analyzed by the *χ*^2^/Fisher exact test. To compare the difference between the steroid group and the PRP group over time, the generalized estimating equation was used to estimate the time × treatment interaction. A negative interaction represents the ability of the generalized estimating equation in indicating the difference between the steroid and PRP groups over time. If the interaction existed, the Mann–Whitney *U* test can be used to compare the difference between both groups at the same time point. The Friedman test was used to evaluate the difference between different time points within one group, while the post hoc test was used to compare the data between preoperation and postoperation in one group. The Wilcoxon signed-rank test was used for paired sample in one group. The difference is considered statistically significant when bilateral *α* = 0.05, *P* < 0.05.

## 3. Results

### 3.1. Patient Characteristics

There were 124 patients who completed follow-ups over the period of 1 year with 63 patients in the steroid group and 61 patients in the PRP group. There was no statistically significant difference in age and gender between both groups (*P* > 0.05) (Table 1). Besides, no statistically significant differences were found in VAS, PPTs, F-wave rates and latency, ODI, and physical function (PF) and bodily pain (BP) domains of SF-36 between the two groups before operation (*P* > 0.05) (Table 1).

### 3.2. PRP Longitudinal Data

The PRP group consisted of 61 participants who were enrolled and completed the follow-ups over 1 year. There was no significant difference in terms of VAS, PPTs, ODI, and the PF and BP domains of SF-36 in 1 week postoperation compared to corresponding basal values (median (1st-3rd quartiles); 6.0 (6.0-7.3) vs. 5.0 (5.0-6.0), *P* = 0.887; 580.60 kPa (557.92-601.01) vs. 625.96 kPa (571.53-716.68), *P* = 0.087; 35.0% (26.4-44.0) vs. 27.0% (20.0-40.0), *P* = 0.125; 60.0 (45.0-70.0) vs. 75.0 (60.0-90.0), *P* = 0.284; 41.0 (31.0-51.0) vs. 43.0 (41.0-52.0), *P* = 0.794, respectively). Statistically significant improvements were observed in terms of VAS, PPTs, ODI, and the PF and BP domains of SF-36 in 1-month postoperation compared to corresponding basal values (Tables 2 and 3). The median (1st-3rd quartiles) VAS was 6.0 (6.0-7.3), which decreased significantly to 3.0 (3.0-4.0) 1 month after operation (*P* < 0.001). Over the same period of time, PPTs had also significantly improved from 580.60 kPa (557.92-601.01) to 707.60 kPa (612.35-780.18) (*P* < 0.001), and ODI reduced from 35.0% (26.4-44.0) to 22.0% (14.25-42.5) (*P* < 0.001). The PF and BP domains of SF-36 had also significantly improved from baseline (60.0 (45.0-70.0) and 41.0 (31.0-51.0), respectively) to 1 month postoperation (88.0 (76.5-95.0), *P* < 0.001; 52.0 (41.0-62.0), *P* < 0.001, respectively). In the PRP group, statistically significant differences were also observed between baseline VAS, PPTs, ODI, and PF and BP domains of SF-36 and the same set of data from 3 months, 6 months, and 1 year postoperation (Tables 2 and 3). F-wave rate was higher after the administration of transforaminal PRP injection, increasing from 82.0% (80.0%-85.0%) to 95.0% (92.0%-100.0%) 1 year after operation (*P* < 0.001). Significant decrease in F-wave latency was observed in the PRP group, where it decreases from 48.7 ms (46.9-49.7) to 45.2 ms (44.5-46.2) 1 year postoperation (*P* < 0.001) (Table 3).

### 3.3. Steroid Longitudinal Data

The steroid group consisted of 63 patients who completed all the follow-up sessions over 1 year. There was no significant difference in terms of VAS, PPTs, ODI, and the PF and BP domains of SF-36 in 1 week postoperation compared to corresponding basal values (median (1st-3rd quartiles); 6.0 (5.0-7.0) vs. 6.0 (5.0-6.0), *P* = 1.000; 598.74 kPa (535.24-607.81) vs. 598.74 kPa (526.17-694.00), *P* = 0.683; 27.0% (21.0-43.0) vs. 23.0% (20.0-40.0), *P* = 0.645; 65.0 (55.0-80.0) vs. 70.0 (60.0-90.0), *P* = 0.152; 41.0 (41.0-52.0) vs. 47.0 (41.0-61.0), *P* = 1.000, respectively). Significant improvement was observed from baseline VAS, PPTs, ODI, and PF and BP domains of SF-36 to the same set of data from 1 month postoperation (Tables 4 and 5). During this period of time, VAS decreased from 6.0 (5.0-7.0) to 3.0 (3.0-5.0) (*P* < 0.001); PPTs increased from 598.74 kPa (535.24-607.81) to 598.74 kPa (526.17-694.00) (*P* < 0.001); ODI significantly decreased from 27.0% (21.0-43.0) to 18.0% (12.0-29.0) (*P* < 0.001), whereas the PF and BP domains of SF-36 all improved statistically from baseline (65.0 (55.0-80.0) and 41.0 (41.0-52.0), respectively) to 1 month postoperation (88.0 (75.0-95.0), *P* < 0.001; 52.0 (41.0-72.0), *P* = 0.004, respectively). Statistical differences were found between baseline VAS, PPTs, ODI, and PF and BP domains of SF-36 in the steroid group and data obtained from 3 months, 6 months, and 1 year postoperation (Tables 4 and 5). The F-wave rate had significantly increased from 82.0% (80.0-95.0) to 95.0% (90.0-100.0) 1 year postoperation (*P* < 0.001), while the F-wave latency had significantly decreased from 48.9 ms (47.8-50.8) to 45.2 ms (43.6-46.3) 1 year post operation (*P* < 0.001) (Table 5).

### 3.4. Intergroup Differences

**Figure 3:**
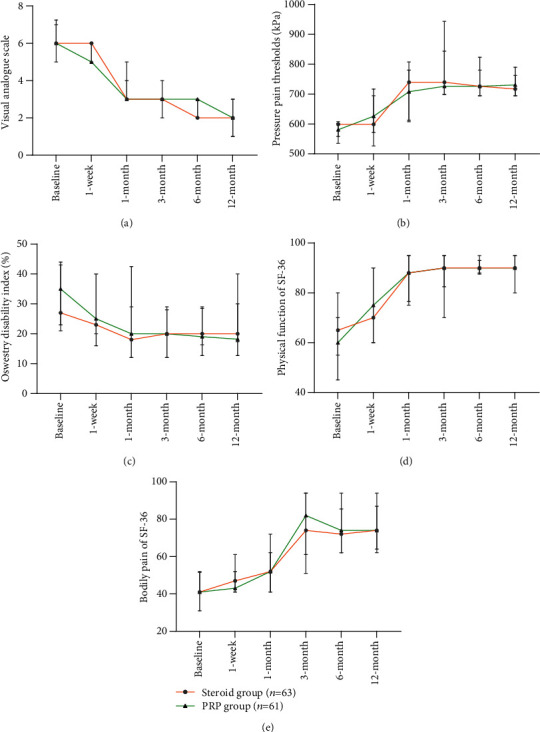
Comparison of patient-reported outcomes between the PRP (*n* = 61) and steroid (*n* = 63) groups over time (median, quartile). There was no significant difference in the (a) visual analogue scale, (b) pressure pain thresholds, (c) Oswestry disability index, and (d) physical function and (e) bodily pain domains of the 36-item short form health survey (SF-36) between the PRP group and the steroid group during follow-ups over the course of one year. The error bars represent the 1st quartile and the 3rd quartile.

**Figure 4:**
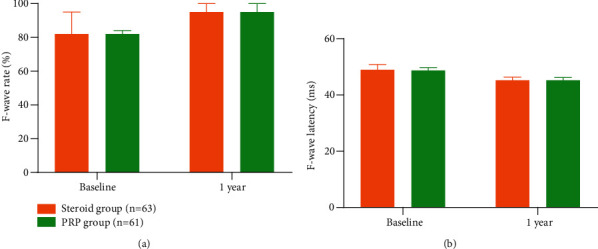
Comparison of F-wave rate and latency between the PRP (*n* = 61) and steroid (*n* = 63) groups. No significant difference was found in terms of F-wave (a) rate and (b) latency between the PRP group and the steroid group both before and after operation. The error bars represent the 3rd quartile.

During the 1-year follow-up period in this study, both the PRP and steroid groups demonstrated obvious improvements in terms of VAS score (Figure 3(a)), PPTs (Figure 3(b)), F-wave rate (Figure 4(a)) and latency (Figure 4(b)), ODI (Figure 3(c)), and the PF (Figure 3(d)) and BP (Figure 3(e)) domains of SF-36. Anyhow, intergroup differences during this 1-year follow-up period were not found to be significant in all tests involved (Figures 3 and 4).

### 3.5. Safety

No complications or adverse effects were reported after the ultrasound-guided transforaminal injection of PRP or steroid during the 1-year follow-up period.

## 4. Discussion

This study shows that ultrasound-guided transforaminal injection of both PRP and steroid leads to the significant improvement in the aspects of pain relief, nerve repair, spinal function, and life quality. Furthermore, the outcome after one whole year of follow-up has proven that these improvements stay effective for long term. Besides, no complications or side effects were found during any of the follow-ups.

Results from this study are in accordance with a series of clinical studies which have described the effectiveness of epidural PRP injections for treating lumbar disc herniation with radicular pain [19–21]. In a nonrandomized comparative trial performed by Bise et al. on 60 patients with lumbar radicular pain in 2020, the CT-guided epidural PRP injection therapy was shown to cause significant pain reduction and functional improvement, which were measured using the numerical rating scale (NRS) and the Oswestry disability index (ODI). The effects of PRP injection were sustained for 6 weeks with no complications reported [19]. Centeno et al. investigated the efficacy of C-arm fluoroscopy-guided epidural platelet lysate injections in patients with lumbar radicular pain and found significant improvement in pain and function from baseline data to data obtained throughout the 2 years of follow-up period, suggesting the potential of PRP as a promising alternative to epidural steroids [20]. In this study, the PF and BP domains from SF-36 showed significant improvement at 1 month after operation and persisted for at least one year in the PRP group. However, there is no statistically significant difference between the PRP group and the steroid group in terms of the PF and BP domains of SF-36. In a randomized, controlled, and double-blinded study performed on 50 patients with complex chronic degenerative spinal pain, Ruiz-Lopez et al. found similar improvement with SF-36 scores measured at 6 months in the PRP group of fluoroscopically guided caudal epidural injection, whereas the steroid group only showed improvement in the BP of SF-36 [22]. Variation in routes of epidural injection and the type of degenerative spinal pain may attribute to these differences in the results.

F-wave measurement helps to assess conduction of impulse along the peripheral motor nerve, including most of its proximal segment. This investigation revealed the significant decrease in F-wave latencies and increase of F-wave rate in both the PRP and steroid groups, indicating the nerve repair function of PRP and steroid. Steroid has positive effects on the F-wave in peripheral nerve disorder, such as chronic inflammatory demyelinating polyradiculopathy and Guillain-Barré syndrome. One study reported that local steroid injection could help to decrease the F-wave latency in carpal tunnel syndrome [23]. Although no studies were found to support the claim about the impact of epidural PRP injection on F-wave latency and the rate in lumbar radicular pain, several studies have reported the positive role of PRP in nerve healing and reduction of neuropathic pain [24, 25]. Anjayani et al. investigated the efficacy of PRP injection in leprosy peripheral neuropathy, which shows the effect of PRP on nerve regeneration and improvement of peripheral neuropathy sensibility [24].

In recent years, PRP has been widely used in treating musculoskeletal diseases due to its anti-inflammatory properties and ability in promoting the processes of endogenous healing by delivering a high concentration of growth factors and cytokines [23, 26]. These growth factors, such as vascular endothelial growth factor (VEGF), transforming growth factor *β*-1 (TGF*β*-1), platelet-derived growth factor (PDGF), and insulin-like growth factor-1 (IGF-1), are contained within the *α*-granules of platelets [26–29]. Within 10 minutes after PRP injection, the platelets aggregate and clot at the targeted site with almost 95% of the *α*-granules load being secreted within 1 hour [21]. Studies have shown that these growth factors are effective in promoting proliferation, angiogenesis, and synthesis of extracellular matrix proteins [26, 30–32]. Therefore, the key rationale behind the application of PRP is to increase the concentration of platelets at the targeted sites so that cytokines and GFs may be released. This will consequently allow the regulation of inflammation and immunological responses of tissue healing [21, 33].

In this investigation, sonography was used to guide transforaminal PRP and steroid injection. The outcomes of nerve root block under the guidance of sonography have proven to be similar to those of injections being guided by either computed tomography scan or X-ray [34–36]. Furthermore, sonography provides the advantages of real-time and dynamic observation with high accuracy, safe, convenient, no radiation, and avoidance of nerve or vessel injury.

No major complications and side effects were reported during the 1-year follow-up period in this study. Only one study reported very rare ischemic complications with lumbar epidural steroid injection by the interlaminar route [37]. Meanwhile, hematomas and infection are known to be the main complications of epidural steroid injection [38, 39]. Henceforth, this makes the autologous PRP a possibly safer alternative with low risks of infection and allergy, since PRP is derived from the patient's own blood and due to the presence of antibacterial proteins in platelets [40]. Furthermore, the systemic side effects of steroid can also be avoided with transforaminal PRP injection [41–43].

Nonetheless, there is currently a lack of standard procedure in PRP production for PRP therapy although a vast variety of formulations and techniques for PRP production is available. Other than that, the cost-effectiveness of PRP treatment remains controversial. On the one hand, the cost of PRP in Europe is reported to be about twice as much as the cost of steroid treatment [44]; on the other hand, a study reported that the cost of PRP therapy for treating orthopedic conditions may actually be less in the long run albeit it appearing to be more expensive than steroid injection in the short term [45].

This study was designed to focus on the long-term treatment effect of transforaminal injection of steroid or platelet-rich plasma (PRP) on lumbar disc herniation. Thus, the evaluation time points were mainly set at 1 month, 3 months, 6 months, and 1 year. The limitation of this study was the lack of short-term evaluation time point, including 2 weeks and 3 weeks. It could be further explored in future clinical trial.

## 5. Conclusions

This study suggests that ultrasound-guided transforaminal PRP injections yield similar effect as transforaminal steroid injections in treating lumbar disc herniation with radicular pain and that it may be a safer alternative in comparison.

## Figures and Tables

**Table 1:** Demographic characteristics and baseline information of patients.

	Steroid group (*n* = 63)	PRP group (*n* = 61)	*P* value
Age (y, median (1^st^-3^rd^))	56.0 (50.0-59.0)	56.0 (44.5-60.0)	0.910
Female (*N* (%))	26 (41.3)	33 (54.1)	0.153
VAS (median (1^st^-3^rd^))	6.0 (5.0-7.0)	6.0 (6.0-7.25)	0.106
PPTs (kPa, median (1^st^-3^rd^))	598.74 (535.24-607.81)	580.60 (557.92-601.01)	0.703
F-wave rate (%, median (1^st^-3^rd^))	82.0 (80-95)	82.0 (80.0-85.0)	0.161
F-wave latency (ms, median (1^st^-3^rd^))	48.9 (47.8-50.8)	48.7 (46.9-49.7)	0.217
ODI (%, median (1^st^-3^rd^))	27.0 (21.0-43.0)	35.0 (26.35-44.0)	0.193
PF of SF-36 (median (1^st^-3^rd^))	65.0 (55.0-80.0)	60.0 (45.0-70.0)	0.091
BP of SF-36 (median (1^st^-3^rd^))	41.0 (41.0-52.0)	41.0 (31.0-51.0)	0.428

PRP: platelet-rich plasma; 1st-3rd: 1st-3rd quartiles; PPTs: pressure pain thresholds; VAS: visual analogue scale; ODI: Oswestry disability index; SF-36: the 36-item short form health survey; PF: physical function; BP: bodily pain.

**Table 2:** Longitudinal outcomes of pain degree and spinal function for the PRP group over time.

Outcome	Time	PRP group (*n* = 61)	*P* value^#^
VAS (median (1^st^-3^rd^))	Baseline	6.0 (6.0-7.3)	Ref
	1 week	5.0 (5.0-6.0)	0.887
	1 month	3.0 (3.0-4.0)	<0.001
	3 months	3.0 (3.0-3.0)	<0.001
	6 months	3.0 (2.0-3.0)	<0.001
	1 year	2.0 (1.0-3.0)	<0.001
	*P* value over time^↑^	<0.001	

PPTs (kPa, median (1^st^-3^rd^))	Baseline	580.60 (557.92-601.01)	Ref
	1 week	625.96 (571.53-716.68)	0.087
	1 month	707.60 (612.35-780.18)	<0.001
	3 months	725.75 (698.53-843.68)	<0.001
	6 months	725.75 (694.00-823.27)	<0.001
	1 year	730.28 (694.00-789.25)	<0.001
	*P* value over time^↑^	<0.001	

ODI (%, median (1^st^-3^rd^))	Baseline	35.0 (26.4-44.0)	Ref
	1 week	27.0 (20.0-40.0)	0.125
	1 month	22.0 (14.3-42.5)	<0.001
	3 months	20.0 (16.5-29.0)	<0.001
	6 months	20.0 (14.0-29.0)	<0.001
	1 year	19.0 (15.5-30.0)	<0.001
	*P* value over time^↑^	0.001	

PRP: platelet-rich plasma; 1st-3rd: 1st-3rd quartiles; PPTs: pressure pain thresholds; VAS: visual analogue scale; ODI: Oswestry disability index. ^#^*P* value compares difference from baseline using post hoc test or Wilcoxon signed-rank test. ^↑^*P* value indicates significance of overall change over time using the Friedman test.

**Table 3:** Longitudinal outcomes of life quality and nerve function for the PRP group over time.

Outcome	Time	PRP group (*n* = 61)	*P* value^#^
PF of SF-36 (median (1^st^-3^rd^))	Baseline	60.0 (45.0-70.0)	Ref
	1 week	75.0 (60.0-90.0)	0.284
	1 month	88.0 (76.5-95.0)	<0.001
	3 months	90.0 (82.5-95.0)	<0.001
	6 months	90.0 (87.5-93.0)	<0.001
	1 year	90.0 (90.0-95.0)	<0.001
	*P* value over time^↑^	<0.001	

BP of SF-36 (median (1^st^-3^rd^))	Baseline	41.0 (31.0-51.0)	Ref
	1 week	43.0 (41.0-52.0)	0.794
	1 month	52.0 (41.0-62.0)	0.005
	3 months	82.0 (61.0-94.0)	<0.001
	6 months	74.0 (62.0-85.5)	<0.001
	1 year	74.0 (64.0-87.0)	<0.001
	*P* value over time^↑^	<0.001	

F-wave rate (%, median (1^st^-3^rd^))	Baseline	82.0 (80.0-85.0)	Ref
	1 year	95.0 (92.0-100.0)	<0.001

F-wave latency (ms, median (1^st^-3^rd^))	Baseline	48.7 (46.9-49.7)	Ref
	1 year	45.2 (44.5-46.2)	<0.001

PRP: platelet-rich plasma; 1st-3rd: 1st-3rd quartiles; SF-36: the 36-item short form health survey; PF: physical function; BP: bodily pain. ^#^*P* value compares difference from baseline using post hoc test or Wilcoxon signed-rank test. ^↑^*P* value indicates significance of overall change over time using the Friedman test.

**Table 4:** Longitudinal outcomes of pain degree and spinal function for the steroid group over time.

Outcome	Time	Steroid group (*n* = 63)	*P* value^#^
VAS (median (1^st^-3^rd^))	Baseline	6.0 (5.0-7.0)	Ref
	1 week	6.0 (5.0-6.0)	1.000
	1 month	3.0 (3.0-5.0)	<0.001
	3 months	3.0 (2.0-4.0)	<0.001
	6 months	2.0 (2.0-3.0)	<0.001
	1 year	2.0 (1.0-3.0)	<0.001
	*P* value over time^↑^	<0.001	

PPTs (kPa, median (1^st^-3^rd^))	Baseline	598.74 (535.24-607.81)	Ref
	1 week	598.74 (526.17-694.00)	0.683
	1 month	739.36 (607.81-807.39)	<0.001
	3 months	739.36 (698.53-943.47)	<0.001
	6 months	725.75 (694.00-780.18)	<0.001
	1 year	716.68 (694.00-762.04)	<0.001
	*P* value over time^↑^	<0.001	

ODI (%, median (1^st^-3^rd^))	Baseline	27.0 (21.0-43.0)	Ref
	1 week	23.0 (20.0-40.0)	0.645
	1 month	18.0 (12.0-29.0)	<0.001
	3 months	20.0 (12.0-29.0)	<0.001
	6 months	20.0 (16.3-29.0)	<0.001
	1 year	20.0 (17.3-40.0)	<0.001
	*P* value over time^↑^	0.001	

1st-3rd: 1st-3rd quartiles; PPTs: pressure pain thresholds; VAS: visual analogue scale; ODI: Oswestry disability index. ^#^*P* value compares difference from baseline using post hoc test or Wilcoxon signed-rank test. ^↑^*P* value indicates significance of overall change over time using the Friedman test.

**Table 5:** Longitudinal outcomes of life quality and nerve function for the steroid group over time.

Outcome	Time	Steroid group (*n* = 63)	*P* value^#^
PF of SF-36 (median (1^st^-3^rd^))	Baseline	65.0 (55.0-80.0)	Ref
	1 week	70.0 (60.0-90.0)	0.152
	1 month	88.0 (75.0-95.0)	<0.001
	3 months	90.0 (70.0-95.0)	<0.001
	6 months	90.0 (88.0-95.0)	<0.001
	1 year	90.0 (80.0-95.0)	<0.001
	*P* value over time^↑^	<0.001	

BP of SF-36 (median (1^st^-3^rd^))	Baseline	41.0 (41.0-52.0)	Ref
	1 week	47.0 (41.0-61.0)	1.000
	1 month	52.0 (41.0-72.0)	0.004
	3 months	74.0 (51.0-94.0)	<0.001
	6 months	72.0 (62.0-94.0)	<0.001
	1 year	74.0 (62.0-94.0)	<0.001
	*P* value over time^↑^	<0.001	

F-wave rate (%, median (1^st^-3^rd^))	Baseline	82.0 (80.0-95.0)	Ref
	1 year	95.0 (90.0-100.0)	<0.001

F-wave latency (ms, median (1^st^-3^rd^))	Baseline	48.9 (47.8-50.8)	Ref
	1 year	45.2 (43.6-46.3)	<0.001

1st-3rd: 1st-3rd quartiles; SF-36: the 36-item short form health survey; PF: physical function; BP: bodily pain. ^#^*P* value compares difference from baseline using post hoc test or Wilcoxon signed-rank test. ^↑^*P* value indicates significance of overall change over time using the Friedman test.

## Data Availability

Participant original data used to support the findings of this study are available in http://www.medresman.org.cn/pub/cn/proj/search.aspx.

## Abbreviations

PRP: Platelet-rich plasma
PPTs: Pressure pain thresholds
VAS: Visual analogue scale
ODI: Oswestry disability index
PF: Physical function
BP: Bodily pain
SF-36: 36-item short form health survey
GFs: Growth factors
NRS: Numerical rating scale
VEGF: Vascular endothelial growth factor
TGF*β*-1: Transforming growth factor *β*-1
PDGF: Platelet-derived growth factor
IGF-1: Insulin-like growth factor-1.

## Supplementary Materials

Supplementary Materials The supplementary material was the CONSORT 2010 checklist of information to include when reporting a randomized trial.
